# The Role of Adiponectin in Breast Cancer: A Meta-Analysis

**DOI:** 10.1371/journal.pone.0073183

**Published:** 2013-08-22

**Authors:** Li-Yuan Liu, Meng Wang, Zhong-Bing Ma, Li-Xiang Yu, Qiang Zhang, De-Zong Gao, Fei Wang, Zhi-Gang Yu

**Affiliations:** 1 Epidemiology Institute, School of Public Health, Shandong University, Jinan, Shandong, China; 2 Department of Breast Diseases, the Second Hospital of Shandong University, Jinan, Shandong, China; University of South Alabama Mitchell Cancer Institute, United States of America

## Abstract

Published results suggests that high adiponectin level may decrease the risk of breast cancer. However, available evidence on breast cancer is conflicting. Therefore a meta-analysis was performed to assess the association between blood adiponectin and breast cancer risk. PubMed database, Web of Science, Elsevier Science, Springer Link and bibliographies of retrieved articles were searched for epidemiological studies published up to March 2013. Meta-analysis was performed on the combined effect values (OR) as well as standardized mean difference (SMD) including 17 studies. Fixed or random effect pooled measure was selected on the basis of homogeneity test among studies. The publication bias was assessed by the Egger’s regression asymmetry test and Begg’s rank correlation test with Begg’s funnel plot. Subgroup analyses and sensitivity analysis were also performed. A total of 13 studies involving 3578 breast cancer cases and 4363 controls contributed to the OR analysis. The high adiponectin level did not significantly affect breast cancer risk (OR=0.902, 95% CI=0.773–1.053). After excluding articles that were the key contributors to between-study heterogeneity, the OR of high adiponectin level was associated with decreased breast cancer risk (OR=0.838, 95% CI=0.744–0.943). There was a significantly association between high adiponectin level and postmenopausal breast cancer women (OR=0.752, 95%CI=0.604-0.936); and it was not associated with premenopausal breast cancer women (OR=0.895, 95%CI=0.638-1.256). The result of pooled measure on SMD was that the high adiponectin level was associated with decreased breast cancer risk (SMD= -0.348, 95% CI= -0.533--0.614) after excluding articles which were the key contributors to between-study heterogeneity. Our findings indicate that high adiponectin level might decrease the risk of postmenopausal breast cancer. More randomized clinical trials and observational studies are needed to confirm this association with underlying biological mechanisms in the future.

## Introduction

Breast cancer is the most common cancer and the leading cancer-related cause of death among women worldwide, with 1.38 million new cases diagnosed in 2008 and causing 458,000 deaths [1]. Obesity is also a common health problem in modern times, and it has increased globally in recent decades because of reduced physical activity and eating habits found in developed countries. In many industrialized countries, over one-fifth of the adult population is obese, and this proportion is also increasing in developing countries [2]. It is known that obesity has been strongly associated with increased risks, and it has been found to be one of the common factors in breast cancer risk of postmenopausal women [3,4]. Interventions of obesity -related provide an important idea for breast cancer prevention. As important adipokines, adiponectin is considered to be one of the key factors for obesity carcinogenic [5], and it is believed to be an important link of the connection between obesity and breast cancer [6].

A recent hypothesis suggests a major role for adipose tissue in carcinogenesis. During many years, the adipose tissue was only considered as a fat storage of energy. This tissue is now described as an endocrine organ secreting a large range of molecules called adipokines. Among these adipokines, adiponectin may play a major role in cancers. Adiponectin is considered as a protective hormone, as it plays a major role in the regulation of glucose with potent insulin-sensitizing activity affecting the uptake of glucose in the muscle, in lipid homeostasis, and is involved in the pathophysiology of atherosclerosis with anti-inflammatory activity [7]. Numerous studies have shown an inhibitory activity of adiponectin on the proliferation of various cell types, including aortic smooth muscle cells, endothelial cells, and several types of cancer cells [8–10]. In previous epidemiologic studies investigating associations between adiponectin and breast cancer, investigators have assumed that blood adiponectin concentrations are good surrogates of tissue exposure. The studies lead to the hypothesis that this adipokine may be a protective factor against breast cancer [7]. However, to our knowledge, there are no studies that confirm this assumption. More convincing evidence is needed to fully elucidate the exact role of adiponectin in breast cancer, since both its beneficial effects and possible mechanisms remain controversial. Understanding of the mechanisms connecting adiponectin with cancer is expected to be of importance in the development of preventive and therapeutic strategies against cancer [11]. This review covers the recent insights into the role of adiponectin in breast cancer, especially the potential link between adiponectin and breast carcinoma.

## Methods

### Search Strategy

English language papers on research in female and human were considered in this study. A comprehensive search was conducted for relevant articals published up to March 16th, 2013 from the following databases : (1) PubMed; (2) Web of Science; (3) Elsevier Science; (4) Springer Link. Search term (adiponectin or Adipo or APN) and (breast cancer or breast carcinoma or mammary cancer) were used in combination to retrieve the relevant literatures in all these databases. Reference lists of articles were scrutinized to identify additional articles. This systematic review was planned, conducted, and reported in adherence to standards of quality for reporting meta-analysis [12].

### Eligibility Criteria

Studies were included in the meta-analysis if they met the following criteria: (1) the study must be an original epidemiologic study of cohort or case-control; (2) the exposure of interest must be the (serum or plasma) adiponectin detecting in blood samples; (3) the outcome of interest must be breast cancer; (4) the article must report odds ratio (OR) or relative risk (RR), corresponding 95% confidence intervals(CI) or data to calculate these(5); mean and standard deviation (SD) or data provided from which mean and SD could be calculated. Two investigators (Li-yuan Liu and Meng Wang) reviewed all studies independently to identify and determine whether an individual study was eligible for inclusion in this meta-analysis. If there was disagreement between the two investigators about eligibility of the article, it was resolved by consensus with a third reviewer.

### Data Extraction

The following data were extracted from each study: the first author’s last name, publication year, country where the study conducted, study design, number of subjects, mean and SD, ORs and 95%CI (*LL* and *UL*), confounders adjusted for in multivariate analysis. Meanwhile, the study quality was evaluated using the 9-star Newcastle-Ottawa Scale [13], a validated technique for assessing the quality of non-randomized studies in meta-analysis.

### Statistical Analysis

We investigated the association between the blood adiponectin and the risk of breast cancer. Heterogeneity of effect size among studies was assessed by the Cochran’s *Q*-test and estimated the amount of variation due to heterogeneity by calculating the *I* ^2^. *I* ^2^ describes the proportion of total variation attributable to between-study heterogeneity as opposed to random error or chance [14]. If substantial heterogeneity existed(*I* ^2^>50%) [15], the random-effects model (REM) was used to assign the weight of each study according to the DerSimonian-Laird method [16]; Otherwise, the fixed-effects model (FEM) was adopted as the pooling method. Sensitivity analysis was performed to test the robustness of the results of meta-analysis. To explore the sources of heterogeneity, we also conducted the subgroup analysis based on the geographic region of studies and the menopausal status included in the study. We tested for publication bias by means of Egger’s regression asymmetry test [17] and Begg’s rank correlation test with Begg’s funnel plot. An analysis of influence was conducted [18], which describes how robust the pooled estimator was to removal of individual studies. An individual study is suspected of excessive influence if the point estimate of its omitted analysis lies outside the 95% CI of the combined analysis. All statistical analyses were performed with Stata software, version 11.0. All reported probabilities (*P* values) were two-sided ,with *P*<0.05 considered statistically significant except where otherwise specified.

## Results

### Literature Search

**Figure 1:**
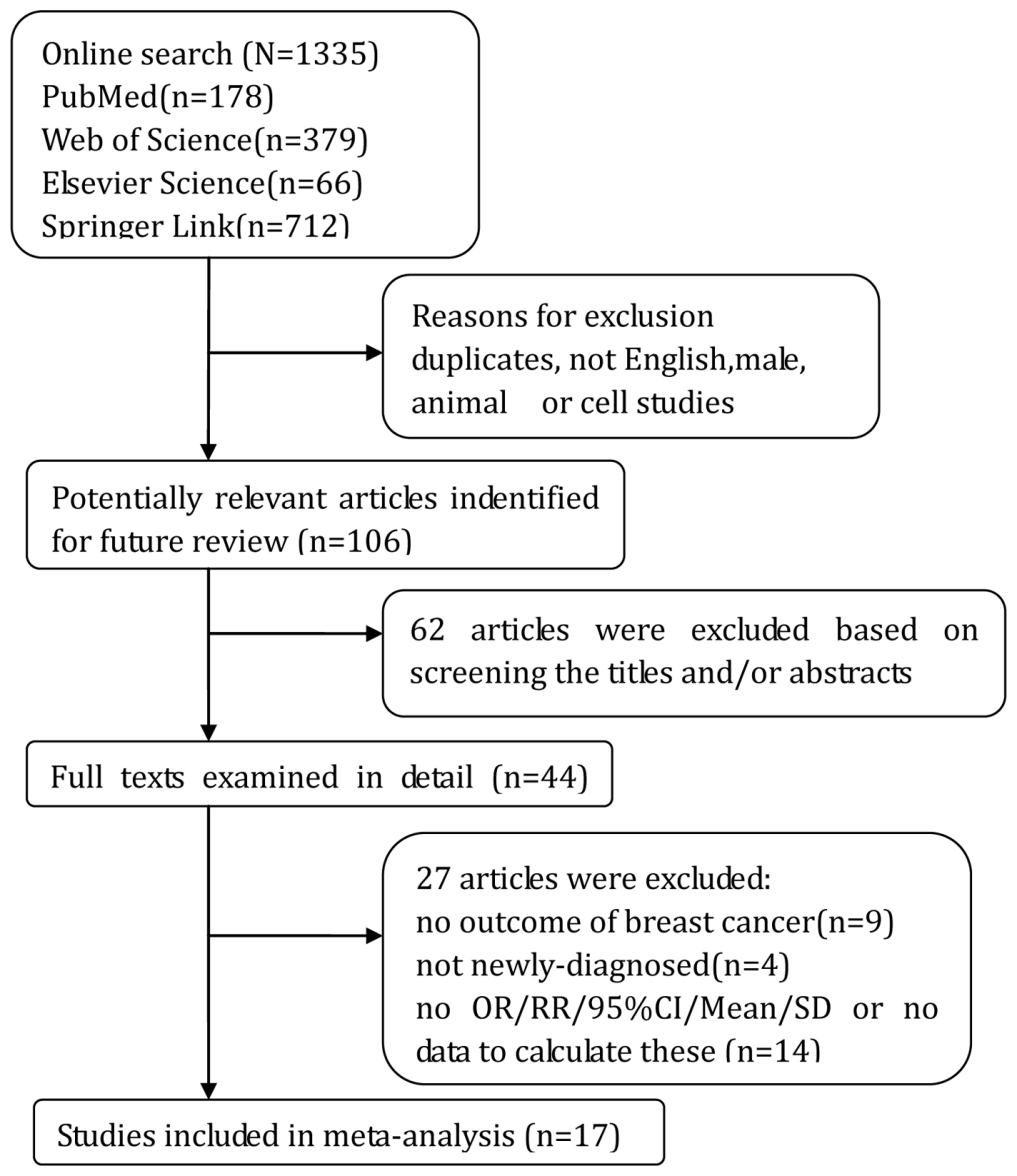
Flow diagram of the study selection process.

The detailed steps of our literature selection are shown in Figure 1. We identified 44 potentially relevant articles concerning adiponectin in relation to breast cancer risk. 27 articles were excluded because of not meeting the eligibility criteria (1), (4) and (5). Finally, 17 studies were available to analysis, 13 articles were included in OR meta-analysis involving 4 nested case-control studies [19–22] and 9 case-control studies [23–31]. With regard to the pooled measure on SMD, 13 articles with 1 nested case-control studies [19] and 12 case-control studies [23,24,26–35]. No cross-sectional or randomized controlled trials (RCT) studies were included based on our eligibility criteria.

### Characteristics of included studies

**Figure 2:**
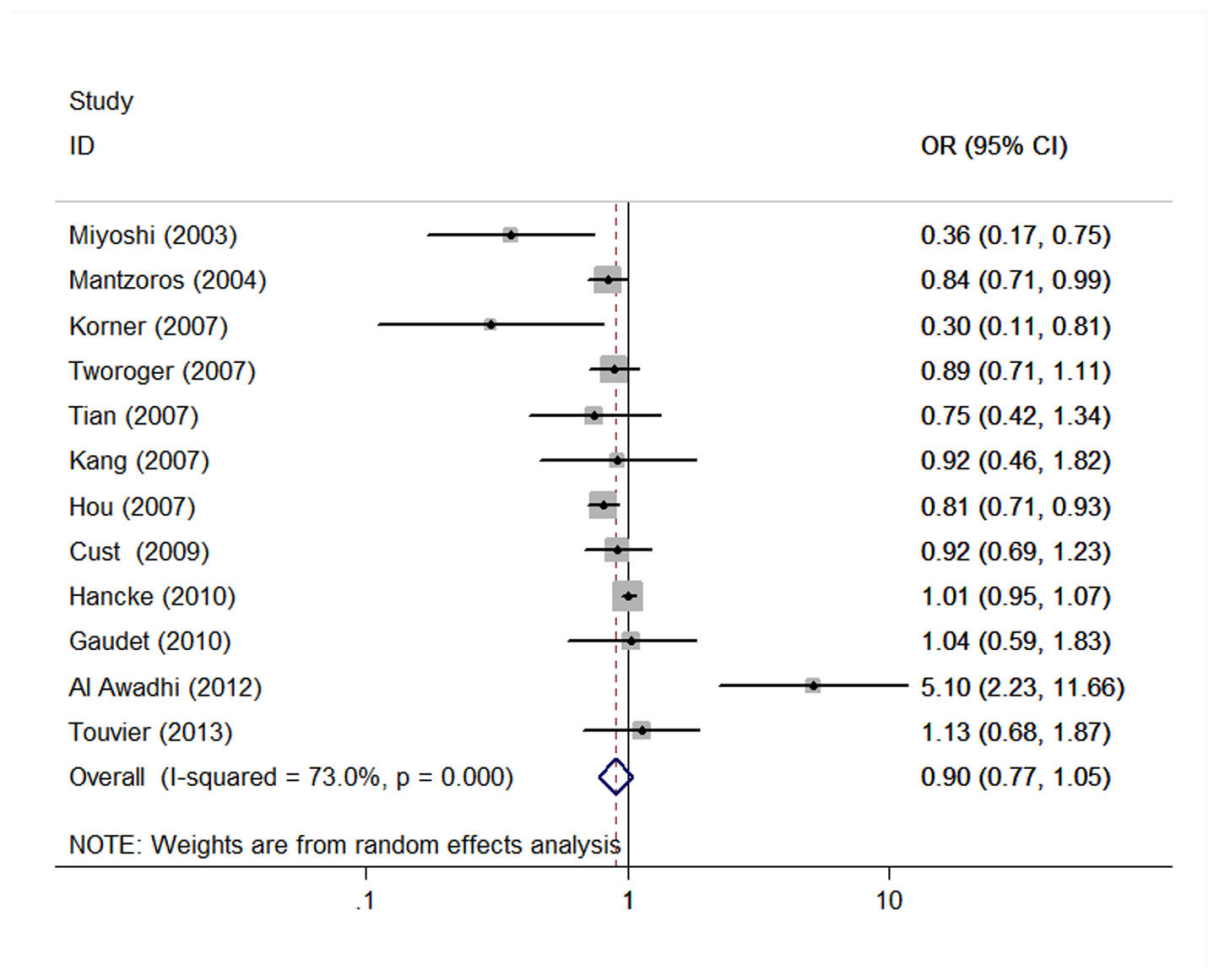
The effect of adiponectin on breast cancer (OR).

We found 13 published articles for the meta-analysis performed on OR, which were published from 2003 to 2013, which outcomes were included involving 3578 cases and 4363 controls. 6 studies in Asia (China, Korea, Kuwait, Malaysia, Japan), 3 studies were conducted in America (USA), 3 studies in Europe (France, Germany, Greece), and 1 study in Australia. Besides, there are 8 researches collected the serum sample [20,24,26–31], 5 researches collected the plasma sample [19,21–23,25]. Most individual studies were adjusted for a wide range of potential confounders, including age, education, BMI, parity, smoking, alcohol, hormone replacement therapy, and so on. The quality score of studies ranged from 7 stars to 9 stars according to the 9-star Newcastle-Ottawa Scale [13]. General characteristics in the published articles in this meta-analysis are shown in Table 1. With respect to the pooled measure on SMD, the studies were eligible for the meta-analysis (8 outcomes for Asia, 3 outcomes for Europe and 2 outcomes for America) were included involving 1407 cases and 1438 controls. A formal test for heterogeneity gave a significant result (*Q*=40.67, *P*<0.001, *I* ^2^=73.0%) and a random-effects model was therefore used. The ORs from studies on blood adiponectin level and breast cancer risk were inconsistent, with both inverse and positive associations reported. The pooled OR of adiponectin in blood was 0.902 (95% CI=0.773–1.053, P<0.01) for breast cancer (Figure 2).

**Table 1:** Characteristics of studies included in the OR meta-analysis.

First author[ref.]	Year	Location	Study design	No. of case/control	OR/RR	LL	UL	Study quality	conclusion^a^	Number of variables adjusted^b^
Touvier M[19]	2013	France	Nested case-control	218/436	1.13	0.68	1.87	8	Ⅰ	1-8
Al Awadhi SA[23]	2012	Kuwait	Case-control	144/77	5.1	2.2	11.5	7	Ⅰ	2,9-13
Shahar S [24]	2010	Malaysia	Case-control	70/138	0.2	0	0.6	7	Ⅱ	2,5,7,14-19
Gaudet MM[20]	2010	USA	Nested case-control	234/234	1.04	0.59	1.83	8	Ⅲ	1,2,20-23
Hancke K[31]	2010	Germany	Case-control	159/41	1.01	0.95	1.07	8	Ⅳ	1,2,17,18,24,
Cust AE[21]	2009	Australia	Nested case-control	561/561	0.92	0.69	1.23	9	Ⅳ	2,17
Hou WK[26]	2007	China	Case-control	80/50	0.81	0.7	0.92	7	Ⅴ	2,25
Kang JH[27]	2007	Korea	Case-control	41/43	0.92	0.46	1.81	7	Ⅳ	1,2
Tian YF[25]	2007	Taiwan	Case-control	244/244	0.75	0.42	1.34	7	Ⅳ,Ⅵ	1,2,24,26-28
Tworoger SS[22]	2007	USA	Nested case-control	1477/2196	0.89	0.71	1.11	9	Ⅳ,Ⅵ	18,23,29-31
Korner A[28]	2007	Greece	Case-control	74/76	0.3	0.11	0.8	8	Ⅱ	18,24,33,33,
Mantzoros C[29]	2004	Greece	Case-control	174/167	0.84	0.71	0.99	7	Ⅱ,Ⅵ	1,2,3,5,7,21,24,32,34
Miyoshi Y[30]	2003	Japan	Case-control	102/100	0.36	0.17	0.74	9	Ⅴ	2,18,20,32

^a ^ Ⅰ The high adiponectin levels were associated with an increased risk for breast cancer. Ⅱ The high adiponectin levels were associated with an decreased risk for breast cancer. Ⅲ There was no association with lower levels of adiponectin and breast cancer risk in postmenopausal women. Ⅳ There was no association with adiponectin and breast cancer risk. Ⅴ The low adiponectin levels were associated with an increased risk for breast cancer. Ⅵ The high adiponectin levels were associated with an decreased risk for breast cancer in postmenopausal women.

^b^ 1 age, 2 Body Mass Index ,3 height, 4 Supplementation of antioxidant vitamins and minerals in intervention group, 5 alcohol intake,6 physical activity,7 smoking status,8 educational level, 9 Insulin, 10 Homeostasis Model Assessment, 11 Leptin, 12 Estradiol,13 Testosterone, 14 working status, 15 age at first pregnancy, 16 Oral contraceptive pill, 17 Hormone Replacement Therapy, 18 family history of breast cancer, 19 lactation, 20 number of births, 21 age at first full term birth, 22 age at menopause, 23 current postmenopausal hormone use,24 menopausal status, 25 waist circumference, 26 date at enrollment, 27 fasting status, 28 waist-to-hip ratio, 29 BMI at age 18, 30 weight change from age 18 to blood draw, 31 history of benign breast disease, 32ages at menarche, 33 nulliparous status, 34 Insulin-like Growth Factor Components.

### Subgroup analysis

**Figure 3:**
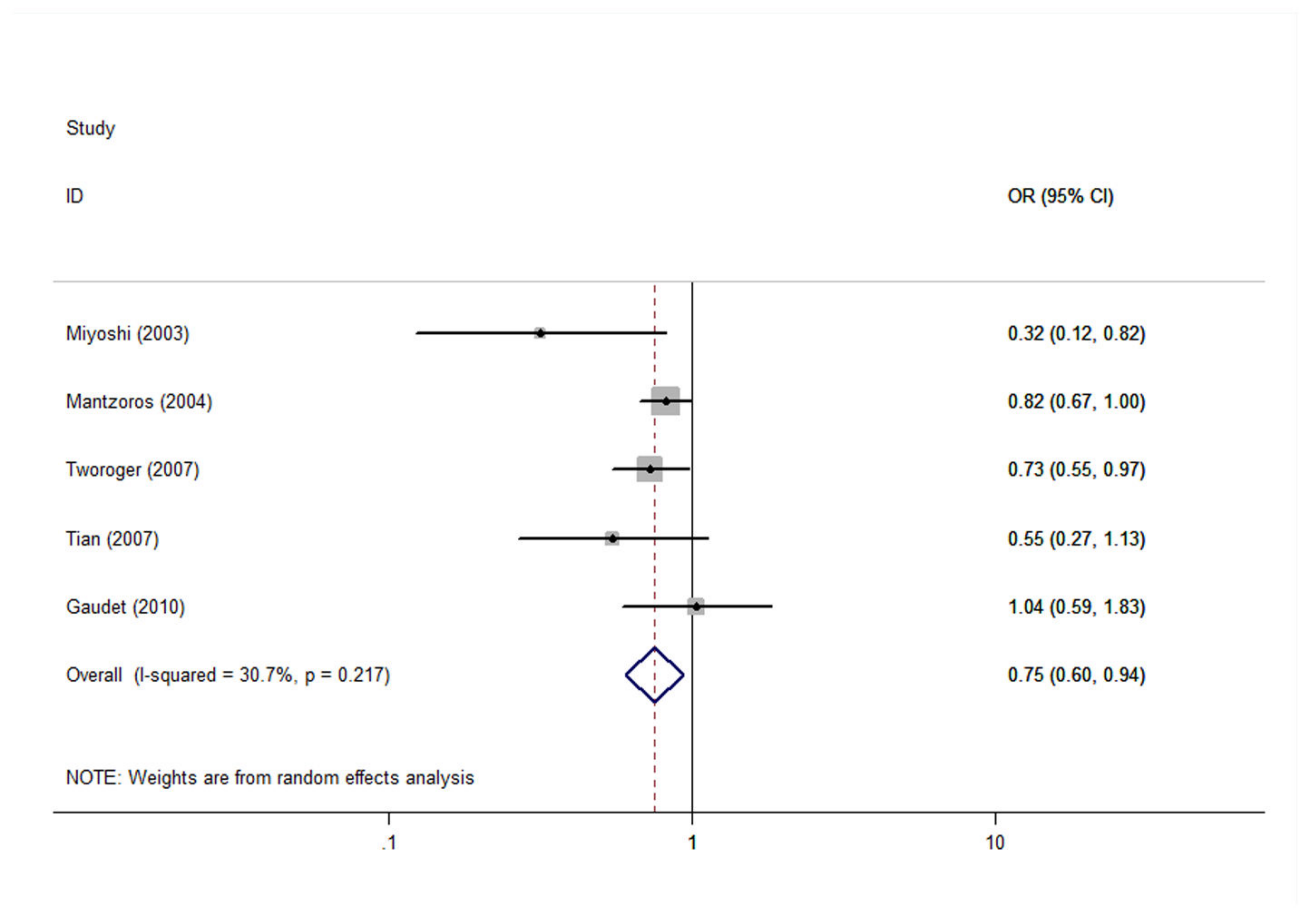
The effect of adiponectin on postmenopausal breast cancer.

**Figure 4:**
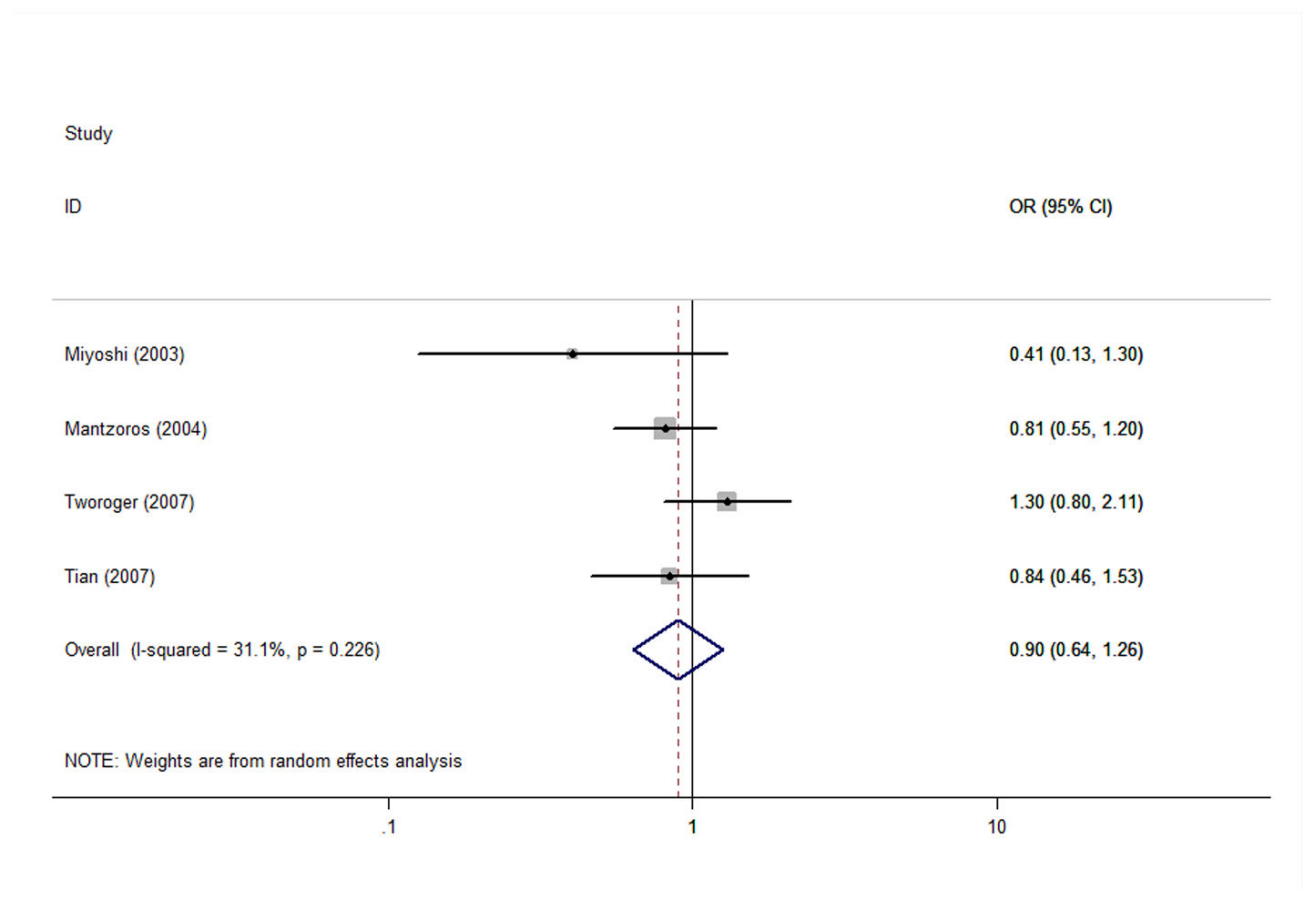
The effect of adiponectin on premenopausal breast cancer.

When we stratified the analysis by the geographic region of studies and the menopausal status, only 5 studies offered relevant available data. The total effect value was 0.75(95%CI=0.60-0.94) (Figure 3) for postmenopausal breast cancer patients, the total effect value was 0.90 (95%CI=0.64-1.26) for premenopausal breast cancer patients (Figure 4). In addition, the total effect value were 0.961 (95%CI=0.540-1.712), 0.910 (95%CI=0.735-1.126), 0.909 (95%CI=0.738-1.119), 0.920((95%CI=0.689-1.228)) in Asia, Europe, America, and Australasia, respectively.

### Sources of heterogeneity and sensitive analysis

To explore the heterogeneity among studies of adiponectin in breast cancer, we performed the sensitive analysis. A sensitivity analysis omitting 1 study at a time and calculating the pooled ORs for the remainder of the studies showed that 2 studies by Al Awadhi SA et al [23] and Hancke K et al [31] may be the key contributor to the between-study heterogeneity. After excluding the 2 studies, there was no study heterogeneity (*Q*=12.22, *P*=0.201, *I* ^2^=26.3%). The pooled OR of adiponectin in blood was 0.838 (95% CI=0.744–0.943, P<0.01) for breast cancer.

### Influence analysis

**Figure 5:**
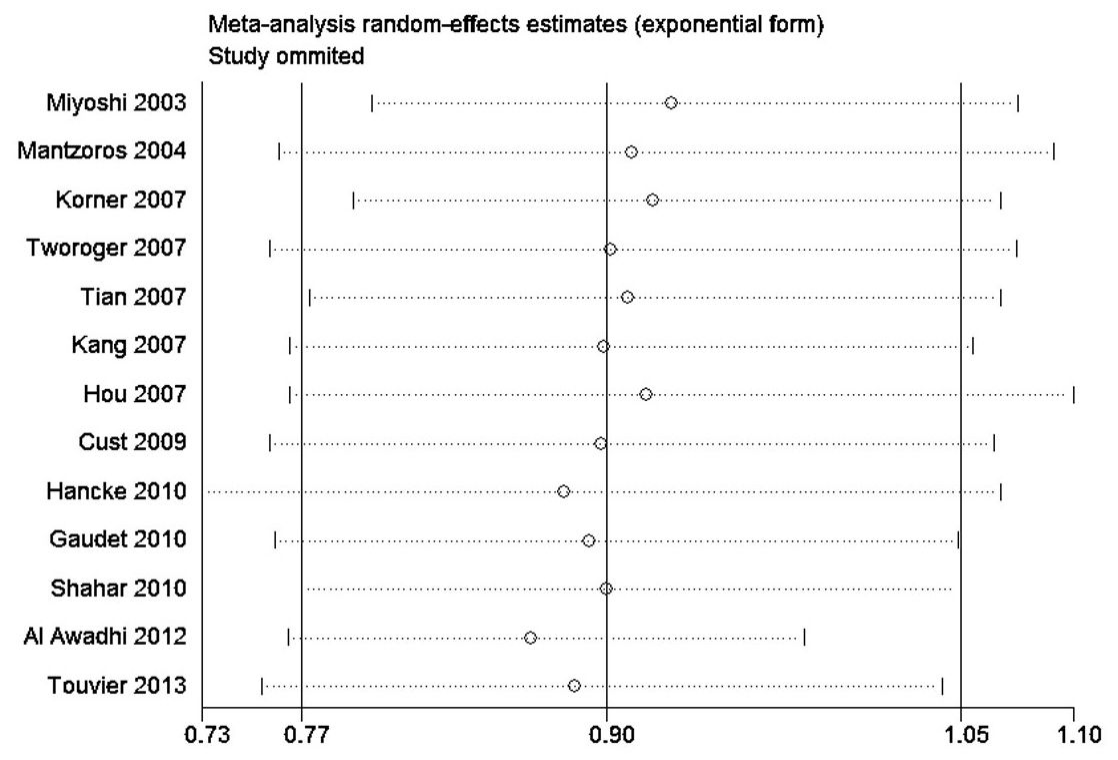
The influence of individual study on the pooled estimate (OR).

It indicated that no individual study had excessive influence the stability of pooled effect except for the study of Al Awadhi SA [23]. A positive association between high adiponectin level and risk of breast cancer in the study (OR = 5.1,95%CI=2.2-11.5) (Figure 5).

### Estimation of publication bias

**Figure 6:**
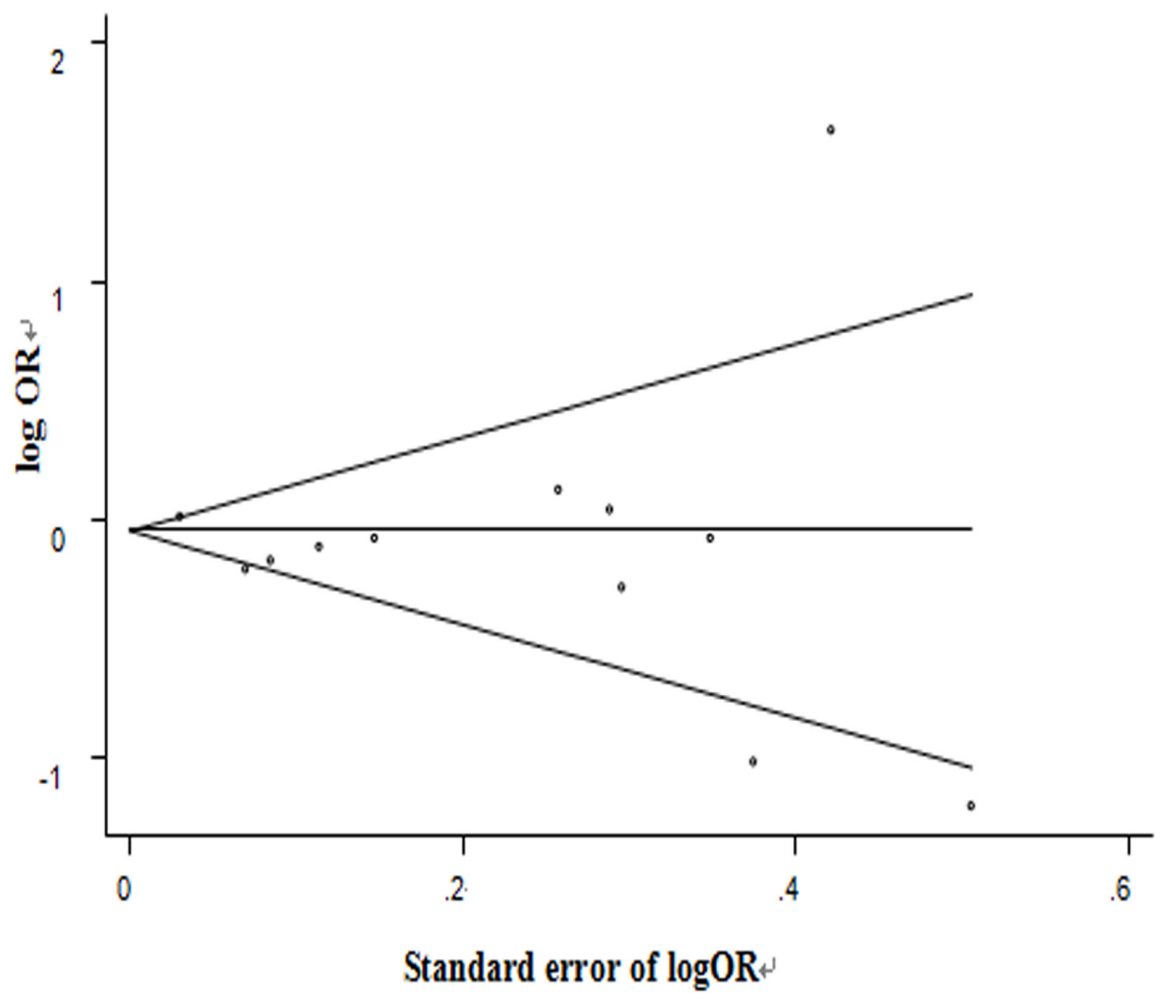
Begg’s funnel plot (with pseudo 95% confidence intervals) to detect any publication bias.

The Begg’s funnel plot showed no striking evidence of publication bias (Figure 6). Neither Egger’s regression asymmetry test (*P*=0.459) nor Begg’s rank correlation test (*P*=1.000) gave a statistically significant result.

### Pooled measure on SMD

**Figure 7:**
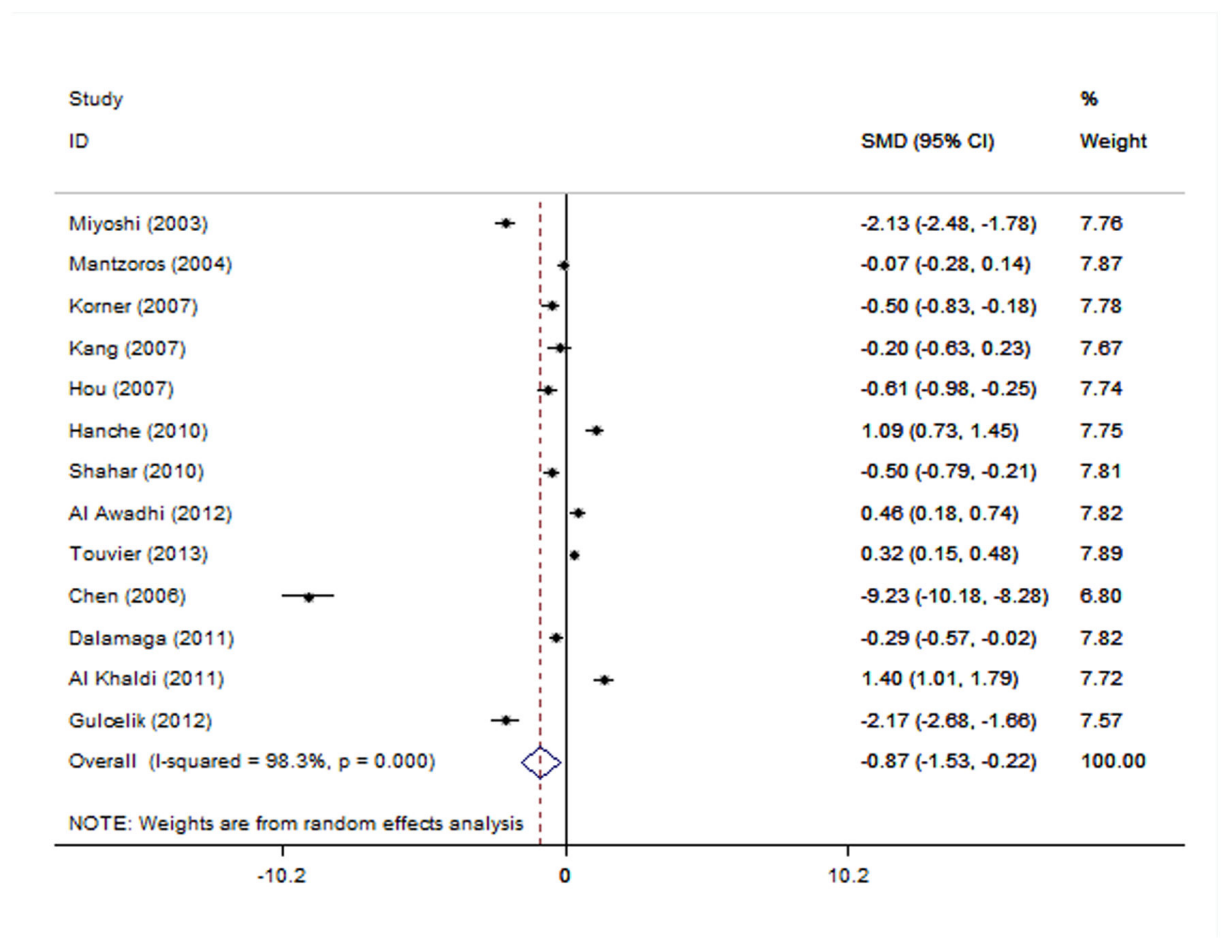
The effect of adiponectin on breast cancer (SMD).

Thirteen studies were available to evaluate the SMD of adiponectin with breast cancer risk. A formal test for heterogeneity gave a significant result (*Q*=720.35 *P*<0.001, *I* ^2^=98.3%), the high adiponectin level was associated with decreased breast cancer risk (SMD= -0.873, 95% CI= -1.529--0.217) (Figure 7). After excluding 7 articles [19,23,30–33,35] which were the key contributors to between-study heterogeneity, the SMD of high adiponectin level was associated with decreased breast cancer risk (*I* ^2^=54.6%, SMD = -0.348, 95% CI= -0.533--0.164). We didn’t stratify the analysis by the menopausal status because only 2 studies offered relevant available data.

## Discussion

As we know the result of a single research may be affected by many factors, in order to reduce the bias and increase the efficiency of the small sample study of statistics, our study in view of the 17 studies to further explore the relationship between blood adiponectin and breast cancer in our meta-analysis. Overall, our results suggest that the blood adiponectin level are no associated with breast cancer risk. However, the subgroup analysis indicated that there is an evident inverse association of adiponectin with breast cancer risk in postmenopausal women. To our knowledge, it was probably the first to attempt to synthesize the existing world literature to evaluate the effect of blood adiponectin on breast cancer.

Adiponectin, a 244-amino acid protein secreted predominantly by white adipose [36], represents the most abundant adipose-tissue protein with insulin-sensitizing, antiinflammatory, and antiatherogenic properties [37,38]. Hypoadiponectinemia is associated not only with insulin resistance, type 2 diabetes, atherosclerosis, and coronary heart disease [39–41], but also with malignancies [42]. Recently, studies have shown that adiponectin is a key mediator in the development and possible progression of several types of obesity-associated cancers [37,43]. Recent studies revealed that the AMP-activated protein kinase (AMPK) may play an important role in the limits of cancer cells proliferation by adiponectin [44], however the mechanism of association is still poorly understood. Understanding the link of adiponectin with cancer might provide potential therapeutic targets, lifestyle amelioration remains the most important component in preventing obesity-related malignancies. Physical exercise, reduction of body weight, a Mediterranean-based diet with consumption of fruits, nuts, coffee, and/or moderate amounts of alcohol present a well-established association with increased adiponectin concentrations and a reduced risk of developing insulin resistance, diabetes, cardiovascular disease, and cancer [45].

The well-established association of obesity and some adipokines with breast cancer risk logically leads to the speculation that adiponectin possibly plays a role in breast cancer development. Although there are a number of studies have shown that adiponectin is closely related to breast cancer, but the inconsistent results from relatively small studies are underpowered to detect the true effect. Our meta-analysis is the appropriate approach to obtain a more definitive conclusion regarding the role of adiponectin on risk of breast cancer. Our meta-analysis, 3578 cases and 4363 controls (OR), 1407 cases and 1438 controls (SMD), allows a much greater possibility of reaching reasonably strong conclusions. The results of our meta-analysis suggest that there is not a significantly positive association of adiponectin with breast cancer. Furthermore, there are no association remains statistically significant in almost all stratified analyses exploring study characteristics and quality factors. Although many studies had shown the high adiponectin level could decrease the risk of breast cancer. Miyoshi et al was the first to report that high adiponectin levels are significantly associated with decreased risk of breast cancer [30]. This potential link was confirmed by several subsequent retrospective case–control observations [25–27,29]. However, some prospective case–control study, in which involving 1,477 breast cancer cases and 2,196 controls demonstrated different results [22]. The outcome showed that there is a lack of strong association between adiponectin and breast cancer risk. Besides in a nested case-control study of 234 postmenopausal women and 234 controls within a cohort of U.S, breast cancer risk was not associated with circulating adiponectin levels independently from other known risk factors [20]. We inferred that the lack of association may be attributed to female sex and hormone levels [46], menstrual status, and BMI etc.

Our present result linked high adiponectin level to a decreased risk only for postmenopausal breast cancer, which were confirmed by a few reviews findings [45,46]. Since the female sex and hormone levels are well-recognized risk factors for breast cancer, the relationship between adiponectin levels and breast cancer risk in pre and post-menopausal women is well investigated. The woman weight gain and excess adiposity are positively associated with breast cancer in postmenopausal women and inversely associated in premenopausal women [47,48]. Miyoshi et al found a fairly robust inverse association between adiponectin and breast cancer risk among both pre- and post-menopausal women [30]. This outcome was supported by Kang et al [27]. However, other groups reported that low adiponectin levels are associated with breast cancer only in post-menopausal women [26,27,29]. Adiponectin concentration has a significant negative correlation with estradiol in postmenopausal but not in premenopausal women [49]. That might help to explicate the divergence, but the mechanism of adiponectin on postmenopausal women and premenopausal women still need to be discussed.

The heterogeneity of between-study is common in meta-analyses, our meta-analysis also showed the significant between-study heterogeneity (OR:I^*2*^ =73.0%, SMD: I ^*2*^ *=* 98.3%). Thus we used sensitivity analysis [50] and subgroup analysis, which aimed to explore the potential important causes of between-study heterogeneity and to reduce the heterogeneity. After the sensitivity analysis using *I* ^*2*^ >50% as the criterion and stratified analysis with study location, our results showed that higher blood adiponectin level could decrease the breast cancer risk, there is a statistical significance with confidence interval not including 1(total OR=0.838, CI=0.744-0.943), which is distinct from the overall result before sensitivity analysis. The omitted article [23,31] may be the key contributor to the between-study heterogeneity. In the SMD analysis, 7 articles were omitted which were half of the study after the sensitivity analysis and the heterogeneity of between-study still over 50% (*I* ^*2*^ =54.6%). Meantime, this suggests we need more large samplings studies to verify our results. Nevertheless, the result of menopausal status group further confirmed previously findings, because there are no data of menopausal status in the omitted study.

In the discussion of the results, some limitations in this meta-analysis should be demonstrated, and the results should be explained with caution. First, although perfect literature searching strategy was used, it was possible that few eligible studies might be missed in this meta-analysis. Second, our meta-analysis was limited to studies published in English. However, we found no evidence of publication bias, although the statistical tests for detecting this had limited power [51], especially for relatively small numbers of studies. Third, the meta-analyses are based on observational studies, and the inherent limitations of such studies may influence our findings. Case–control studies are mainly subject to selection bias, among which the admission rate bias, detection signal bias and time effect bias. For example, in our study the exposure data derived from self-administered questionnaire, in-person or telephone interview. So, there was the possibility that our results had information or selection bias, which could be of concern. Fourth, limitation was the inadequate control of confounding factors in studies since some ORs were calculated with different adjustment. As a kind of cancer was correlated with age, education, BMI, parity, smoking, alcohol, hormone replacement therapy and so on, the inadequate control for these confounders could bias the results and lead to an underestimated or exaggerated effect size. Besides, in the ORs meta-analysis, a single value is assigned to exposure categories, usually characterized by a range. It has been reported [52,53] that allowing for the variation in exposure level within each reported interval could give different results in the estimation of the pooled OR. Finally, considering the small number of studies included in our meta-analysis, it is worth noting that the validity of our publication bias test might be questioned.

In conclusion, findings from this present study indicated that blood adiponectin level was not associated with the risk of breast cancer, yet revealed that there was a weakly protective association of high adiponectin level with postmenopausal breast cancer women. Since potential biases and confounders could not be ruled out completely in this meta-analysis, more randomized clinical trials and large sample size observational studies are needed to confirm this association with underlying biological mechanisms in the future.

## Supporting Information

Checklist S1 (DOC)

Flow Diagram S1 (DOC)
