# Supplementary material for: The Role of Adiponectin in Breast Cancer: A Meta-Analysis
Source: PLoS One. 2013 Aug 22;8(8):e73183. doi: 10.1371/journal.pone.0073183 (PMC3749999; doi:10.1371/journal.pone.0073183)
Supplement: Flow Diagram S1 — (DOC) [file pone.0073183.s002.doc]

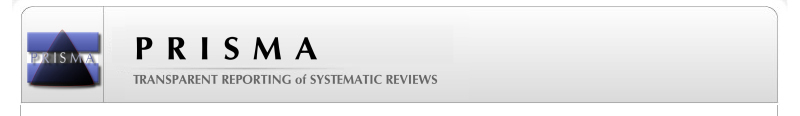
**PRISMA 2009 Flow Diagram**

**Screening**

**Included**

**Eligibility**

**Identification**

Records identified through database searching
(n =1335 )

Additional records identified through other sources
(n =0 )

Records after duplicates removed
(n =152 )

Records screened
(n = 106 )

Records excluded
(n = 62 )

Full-text articles assessed for eligibility
(n = 44 )

Full-text articles excluded, with reasons
(n = 27 )

Studies included in qualitative synthesis
(n =0 )

Studies included in quantitative synthesis (meta-analysis)
(n = 17 )
